# Assessing the accuracy of predictive models for numerical data: Not *r* nor *r^2^*, why not? Then what?

**DOI:** 10.1371/journal.pone.0183250

**Published:** 2017-08-24

**Authors:** Jin Li

**Affiliations:** National Earth and Marine Observations, Environmental Geoscience Division, Geoscience Australia, Canberra, Australian Capital Territory, Australia; China Agricultural University, CHINA

## Abstract

Assessing the accuracy of predictive models is critical because predictive models have been increasingly used across various disciplines and predictive accuracy determines the quality of resultant predictions. Pearson product-moment correlation coefficient (*r*) and the coefficient of determination (*r*^2^) are among the most widely used measures for assessing predictive models for numerical data, although they are argued to be biased, insufficient and misleading. In this study, geometrical graphs were used to illustrate what were used in the calculation of *r* and *r*^2^ and simulations were used to demonstrate the behaviour of *r* and *r*^2^ and to compare three accuracy measures under various scenarios. Relevant confusions about *r* and *r*^2^, has been clarified. The calculation of *r* and *r*^*2*^ is not based on the differences between the predicted and observed values. The existing error measures suffer various limitations and are unable to tell the accuracy. Variance explained by predictive models based on cross-validation (*VEcv*) is free of these limitations and is a reliable accuracy measure. Legates and McCabe’s efficiency (*E*_*1*_) is also an alternative accuracy measure. The *r* and *r*^2^ do not measure the accuracy and are incorrect accuracy measures. The existing error measures suffer limitations. *VEcv* and *E*_*1*_ are recommended for assessing the accuracy. The applications of these accuracy measures would encourage accuracy-improved predictive models to be developed to generate predictions for evidence-informed decision-making.

## Introduction

Predictive models have been increasingly used to generate predictions across various disciplines in the environmental sciences in parallel to the recent advancement in data acquisition, data processing and computing capabilities. Accuracy of the predictive models is critical as it determines the quality of their predictions that form the scientific evidence for decision-making and policy. Therefore, it is important to correctly assess the predictive accuracy. Many accuracy/error measures have been developed to assess the accuracy of predictive models, including correlation coefficient (*r*) and the coefficient of determination (*r*^2^) for numerical data [1–3]. However, it has been advised that *r* and *r*^2^ should not be used as a measure to assess the accuracy of predictive models for numerical data because they are biased, insufficient or misleading [4–10]. It has been further advised that *r* is a measure of correlation, not accuracy [11].

Despite the advice above, *r* and *r*^2^ have been used as predictive accuracy measures in various disciplines in numerous studies and have even been used as accuracy measures in some computing programs/software. Furthermore, *r* and *r*^2^ are among the most widely used measures to assess model performance in many disciplines [2–5,12–14]. Their wide application in assessing predictive accuracy could be resulted from many reasons, such as that: 1) although *r* was found to be a biased measure of predictive accuracy, it was suggested as a measure of potential skill [7]; 2) the differences between the predicted values and the observed values of validation samples are sometimes termed residuals [2], but they are not the residuals that *r* and *r*^2^ are usually applied to [15,16]; 3) *r* and *r*^2^ were proven to be a component of mean square error (*MSE*) [17], and hence a component of root MSE (RMSE) that is one of the most commonly used error measures in the environmental sciences [6]; 4) a weighted *r* was also proposed to alleviate the problem associated with *r* [3]; 5) the advice above were based on computed, modelled or predicted values that were sometimes referred to as fitted values [5,18] which were used to derive *r* and *r*^2^ [15,16]; and 6) no solid evidence was provided to support the advice, although *r* and *r*^2^ were proven to biased [9,10]. Consequently, the advice becomes less convincing and has played little role in preventing people from using *r* and/or *r*^2^ to assess the accuracy of predictive models.

This study aims to 1) clarify relevant confusions about *r* and *r*^2^ and illustrate why they are incorrect measures of predictive accuracy, 2) demonstrate how they are misleading when they are used to assess the accuracy of predictive models, and 3) justify what should be used to assess the accuracy.

## Methods

In this study, *r* was assumed to be the most often used Pearson product-moment correlation coefficient, and *r*^2^ was the coefficient of determination. In fact, *r* is the same as the *r* in *r*^2^ when *r* is positive, which is often the case for predictive modelling. To avoid any confusion, in this study relevant concepts are defined as below:

1. the predicted values (y) were the values obtained from predictive models based on a validation method,
2. the observed values (x) were the values of validation samples,
3. fitted line was based on y and x and was assumed to be linear with a certain slope and an intercept, and
4. fitted values were derived based on the fitted line.

### 2.1. Scenarios simulated for why *r* and *r*^2^ are incorrect measures of predictive accuracy

**Fig 1:**
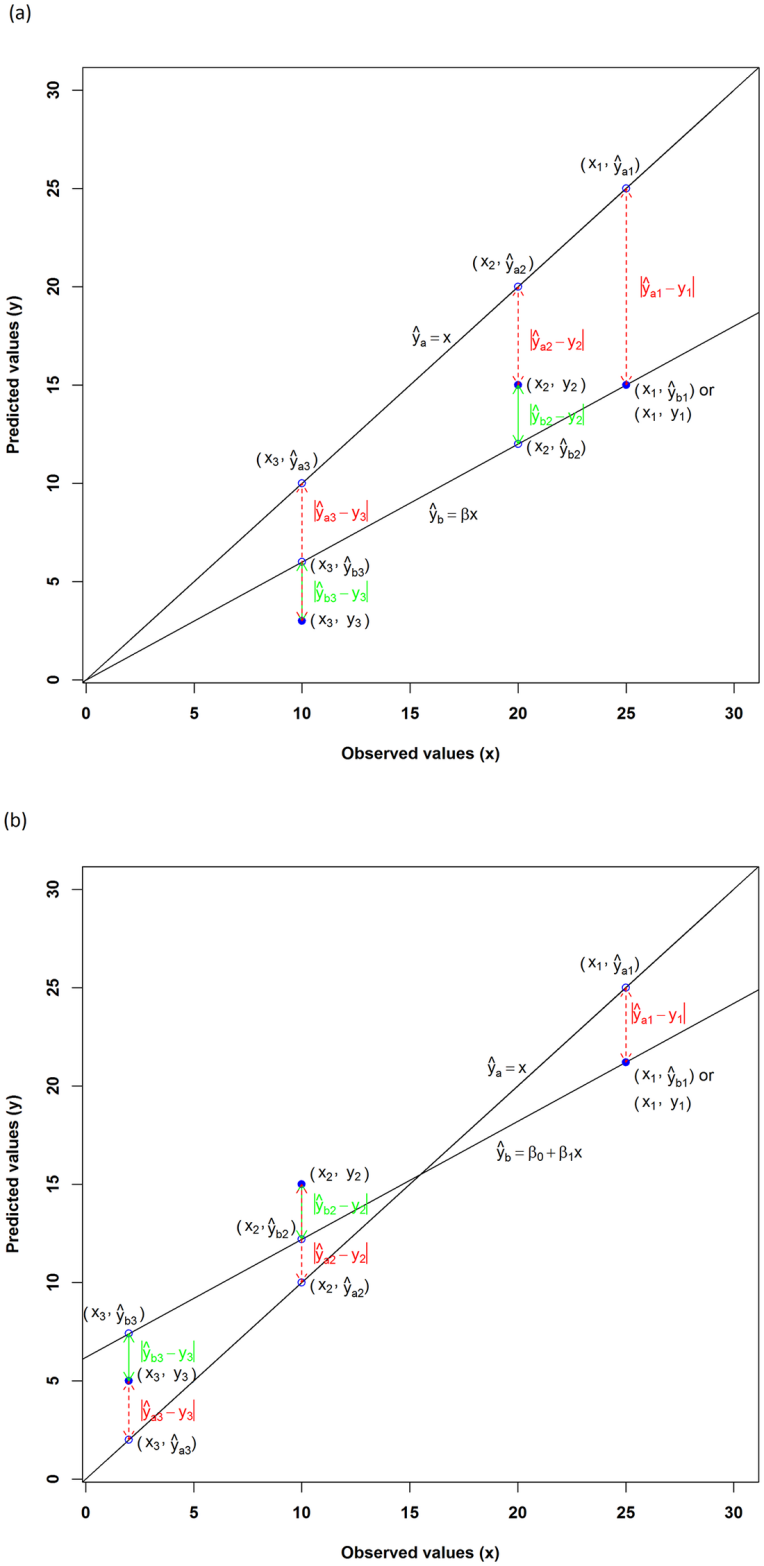
The relationship of the observed values (x) and predicted values (y), where fitted line ŷ_a_ = x, suggesting y and x are perfectly matched. **a**) the fitted line ŷ_b_ = *β*x deviates from ŷ_a_ = x by (1 − *β*)x; and **b**) the fitted line ŷ_b_ = *β*_*0*_ *+ β*_*1*_x deviates from ŷ_a_ = x by (1 − *β*_*1*_)x − *β*_*0*_. For each pair of predicted and observed values (i.e., (x_1_, y_1_), (x_2_, y_2_) and (x_3_, y_3_)), the green lines represent the distance between the fitted and predicted values used for calculating *r*, and the red dashed lines represent the distance between the predicted and observed values.

The relationship between y and x could vary with studies [4,13,19–21]. It was expected to be linear with a slope of 1 and an intercept of 0 (i.e., ŷ_a_ = x, where ŷ_a_ was the fitted values based on y and x, and was equal to y) if a perfect match between y and x was obtained (Fig 1a and 1b). Any predictions deviating from y = x line were not accurate and contained certain errors. The following two scenarios were used to demonstrate why *r* is an incorrect measure of predictive accuracy as they closely represented the reality above. Scenario 1: the fitted values based on y and x were derived from ŷ_b_ = βx (Fig 1a); and scenario 2: the fitted values were derived from ŷ_b_ = β_0_ + β_1_x (Fig 1b). They deviated from the perfect match: ŷ_a_ = x.

In each scenario, we considered four situations (Fig 1a and 1b), i.e., predicted values were:

1. on the fitted line ŷ_a_ = x;
2. on the fitted line ŷ_b_ = βx or ŷ_b_ = β_0_ + β_1_x;
3. above the fitted line ŷ_b_ = βx or ŷ_b_ = β_0_ + β_1_x; and
4. below the fitted line ŷ_b_ = βx or ŷ_b_ = β_0_ + β_1_x.

### 2.2. Scenarios simulated for how *r* and *r*^2^ are misleading

In reality, the slope often deviates away from 1 for y and x; and the intercept also usually deviates from zero. To quantitatively prove how *r* is misleading in assessing the accuracy of predictive models, four scenarios were simulated (S1 Fig):

1. x and y were perfectly linearly related with an intercept of 0, i.e., y = βx (Panel A in S1 Fig);
2. x and y were perfectly linearly related with intercepts changing with their associated slopes, i.e., y = β_0_ + β_1_x (Panel B in S1 Fig);
3. as the first scenario, but with certain noise (ε) in y, i.e., y = βx + ε (Panel C in S1 Fig); and
4. as the second scenario, but with ε in y, i.e., y = β_0_ + β_1_x+ ε (Panel D in S1 Fig).

The first two scenarios were the extensions of the scenarios presented in Fig 1a and 1b, where the predictions matched the observations well and their relationship was assumed to be perfectly linear, but with a slope deviating from 1 and with or without intercepts respectively. They were largely ideal and only used to conveniently illustrate relevant issues associated with *r*.

The last two scenarios more closely reflected the reality [19,20], particularly the last scenario, because predictions were usually noisy and quite often the smaller observed values were predicted larger and the larger observed values were predicted smaller [4,13,21].

It was argued that measures using squared values are more sensitive to data variation or sample size than measures using the absolute values [8,22,23]. To test whether predictive accuracy measure depends on sample size and data variation, the last scenario above was further extended, where the predicted values were with different sample sizes and with noise of different data variations.

### 2.3. Assessment of predictive accuracy

Predictive accuracy should be measured based on the difference between the observed values and predicted values. However, the predicted values can refer to different information. Thus the resultant predictive accuracy can refer to different concepts. The predicted values quite often refer to the values that were predicted or modelled based on training samples [18]; and the resultant accuracy has been termed predictive accuracy in various studies. However, this accuracy is essentially measuring how well the model fits the training samples, thus it is not measuring the predictive accuracy. Predictive accuracy can also be based on the differences between the predicted values for, and the observed values of, new samples (e.g., validation samples). This is the predictive accuracy we refer to in this study.

To demonstrate how misleading *r* is, we need to select an appropriate measure as a reference. All mean absolute error (*MAE*) and *MSE* related measures, and variance explained by predictive models based on cross-validation (*VEcv*) use the correct difference [18]. Of these measures, *VEcv* doesn’t share the limitations associated with these error measures according to Li [18], so *VEcv* was selected as a control to assess the predictive accuracy and to compare with *r*. Additionally, *VEcv* was introduced to avoid relevant issues associated with Nash and Sutcliffe’s efficiency [24], G-value [25] and model efficiency [26]; and they are equivalent to *VEcv* if they are based on predictions derived from validation dataset [18]. Although *VEcv* was initially proposed for predictive models based on cross-validation because 10-fold cross-validation would produce more reliable results [27,28], it can be applied to results based on any validation methods or to any new samples besides validation samples.

To select reliable accuracy measure(s) for future studies, some commonly used error and accuracy measures for numerical data were evaluated (Table 1). Two other accuracy measures, Willmott et al.’s refined index of agreement (*d*_*r*_) [29,30] and Legates and McCabe’s (*E*_*1*_) [31], were considered. They were presented in percentage to make their resultant values comparable with *VEcv*.

**Table 1:** The mathematical definitions of relevant measures used in this study [8,15,18,30].

Error/accuracy Measure	Definition*
Mean absolute error (*MAE*)	$\sum_{1}^{n}\left|y_{i}-\hat{y_{i}}\right|/n$
Mean square error (*MSE*)	$\sum_{1}^{n}{\left(y_{i}-\hat{y_{i}}\right)}^{2}/n$
Relative *MAE* (*RMAE*)	$\left(\frac{MAE}{\bar{y}}\right)100\;\left(\%\right)$
Root *MSE* (*RMSE*)	*MSE*^1/2^
Relative *RMSE* (*RRMSE*)	$\left(\frac{RMSE}{\bar{y}}\right)100\;\left(\%\right)$
Standardised *RMSE* (*SRMSE*)	*RMSE/s*
Mean square reduced error (*MSRE*)	*MSE/s*^2^
Variance explained (*VEcv*)	$\left(1-\frac{\sum_{1}^{n}{\left(y_{i}-\hat{y_{i}}\right)}^{2}}{\sum_{1}^{n}{\left(y_{i}-\bar{y}\right)}^{2}}\right)100\;\left(\%\right)$
Legates and McCabe’s (*E*_*1*_)	$\left(1-\frac{\sum_{1}^{n}|y_{i}-\hat{y_{i}}|}{\sum_{1}^{n}\left|y_{i}-\bar{y}\right|}\right)100\;\left(\%\right)$
Willmott et al.’s refined index of agreement (*d*_*r*_)	*E*_*1*_, if *E*_*1*_ > = 0; $\left(\frac{\sum_{1}^{n}|y_{i}-\bar{y}\;|}{\sum_{1}^{n}\left|y_{i}-\hat{y_{i}}\right|}-1\right)100\;\left(\%\right)$, if *E*_*1*_ < 0
Pearson product-moment correlation coefficient (*r*)	$\frac{\sum_{1}^{n}\left(y_{i}-\bar{y}\right)\left(\hat{y_{i}}-\bar{{\hat{y}}_{i}}\right)}{{\left(\sum_{1}^{n}{\left(y_{i}-\bar{y}\right)}^{2}{\left(\hat{y_{i}}-\bar{{\hat{y}}_{i}}\right)}^{2}\right)}^{1/2}}$

* *n*: the number of observations in a validation dataset; *y*_*i*_: the observed value in the validation data; $\hat{y_{i}}$: the predicted value; $\bar{y}$: mean of the observed values; *s*: standard deviation of the observed values; and $\bar{{\hat{y}}_{i}}$: mean of the predicted value.

For the first and third scenarios, the range of slope was selected to be between 0.1 and 1.2. This choice was to ensure that the range of *VEcv* for the simulated scenarios covers a reasonable range of *VEcv* because the *VEcv* of predictive models was found to be ranging from -153% to 97% based on 296 applications [18] and also to ensure the results can be well illustrated because when the slope was below 0.1, *VEcv* was getting quadratically lower and would distort the illustration; moreover, practically the slope would usually be below 1.2. For the second and last scenarios, the range of slope was selected to be between 0 and 1.2; and setting the slope to be 0 was to simulate when the global mean was used as predictions [18]. All simulation work was implemented in R 3.2.3 [32].

Since this study is based on simulated data only that can be produced using the information provided in this section, no further data are used and available. All relevant R functions and R scripts used for the simulations and subsequent plotting in this study are stored as ‘Measures-of-predictive-errors-and-accuracy-for-PONE-Supporting-information-2.R’ at: https://github.com/jinli22/Not-r-nor-r2.

## Results and discussion

### 3.1. Why *r* and *r*^2^ are incorrect measures of predictive accuracy

When *r* is used to assess the predictive accuracy based on y and x, the relationship between y and x is usually assumed to be linear with a slope significantly larger than 0 and an intercept of any reasonable value. It measures the residuals that are the difference between y and the fitted values that are derived from y and x [15,16]. Its calculation is based on the departures of y from the fitted values, which is essentially a measure of the goodness-of-fit between y and x. Therefore, *r* is not a measure of predictive accuracy. Neither is *r*^2^ because the *r* in *r*^2^ is the same as *r*. The key confusion is that the fitted values have been mistakenly used as x, which is illustrated below.

When *r* is applied to the simulated situations (Fig 1), its calculation is essentially determined by the error sum of squares (i.e., Σ(y– ŷ_b_)^2^) as detailed in Crawley [33], where ŷ_b_ is the fitted values based on the equations as depicted in Fig 1a and 1b and explained below.

1. In the first situation, when the predicted values were on the fitted line ŷ_a_ = x, x and y were equal. The difference between x and y and between y and fitted values were 0.
2. In the second situation, when the predicted values were on the fitted line ŷ_b_ = βx or ŷ_b_ = β_0_ + β_1_x, x and y were matched well proportionally. For example, for an observed value x_1_, with a predicted value y_1_, the difference used for calculating *r* was |y_1_ − ŷ_b1_| and was still 0, where ŷ_b1_ was the corresponding fitted value of x_1_ on ŷ_b_ = βx or ŷ_b_ = β_0_ + β_1_x. However, the real difference between the observed and predicted values is |x_1_ –y_1_| that can be expressed as |ŷ_a1_ –y_1_| given that x_1_ = ŷ_a1_, where ŷ_a1_ was the corresponding value of x_1_ on ŷ_a_ = x.
3. In the third situation, when a predicted value was above the fitted line ŷ_b_ = βx or ŷ_b_ = β_0_ + β_1_x, the predicted value was higher than the fitted value ŷ_b_. For example, for an observed value x_2_, with a predicted value y_2_, the difference used for calculating *r* was |ŷ_b2_– y_2_ |, where ŷ_b2_ was the corresponding fitted value of x_2_ on ŷ_b_ = βx or ŷ_b_ = β_0_ + β_1_x. However, the real difference is |x_2_ –y_2_| that can be expressed as |ŷ_a2_ –y_2_| given that x_2_ = ŷ_a2_, where ŷ_a2_ was the corresponding value of x_2_ on ŷ_a_ = x.
4. In the final situation, when a predicted value was below the fitted line ŷ_b_ = βx or ŷ_b_ = β_0_ + β_1_x, the predicted value was lower than the fitted value ŷ_b_. For example, for an observed value x_3_, with a predicted value y_3_, the difference used for calculating *r* was |ŷ_b3_ –y_3_|, where ŷ_b3_ was the corresponding fitted value of x_3_ on ŷ_b_ = βx or ŷ_b_ = β_0_ + β_1_x. However, the real difference is |x_3_ –y_3_| that can be expressed as |ŷ_a3_ –y_3_| given that x_3_ = ŷ_a3_, where ŷ_a3_ was the corresponding value of x_3_ on ŷ_a_ = x

It is clear that *r* can only be used to assess the predictive accuracy when y and x are equal and perfectly matched, where the fitted values are equal to y. In all other cases, i.e., when the intercept is not zero and/or the slope deviates from 1 or y and x are not well matched (Fig 1a and 1b), the fitted values are used to calculate *r*, and the calculation of *r* is not based on the difference between the predicted values and observed values. Hence *r* is not a correct measure of predictive accuracy. Neither is *r*^2^ given that *r* in *r*^2^is the same as *r*. Although several studies have pointed that *r* and *r*^2^ are biased, insufficient and misleading measures of predictive accuracy [4–10], no study has demonstrated that their calculations were not based on the difference between the predicted values and observed values. On the basis of above demonstration, it can be concluded that *r* and *r*^2^ are incorrect measures of predictive accuracy.

### 3.2. How are *r* and *r*^2^ misleading in assessing the accuracy of predictive models?

Despite the illustration above, it is still unclear how misleading *r* and *r*^2^ are when they are used to assess the predictive accuracy. This needs to be quantitatively evidenced.

**Fig 2:**
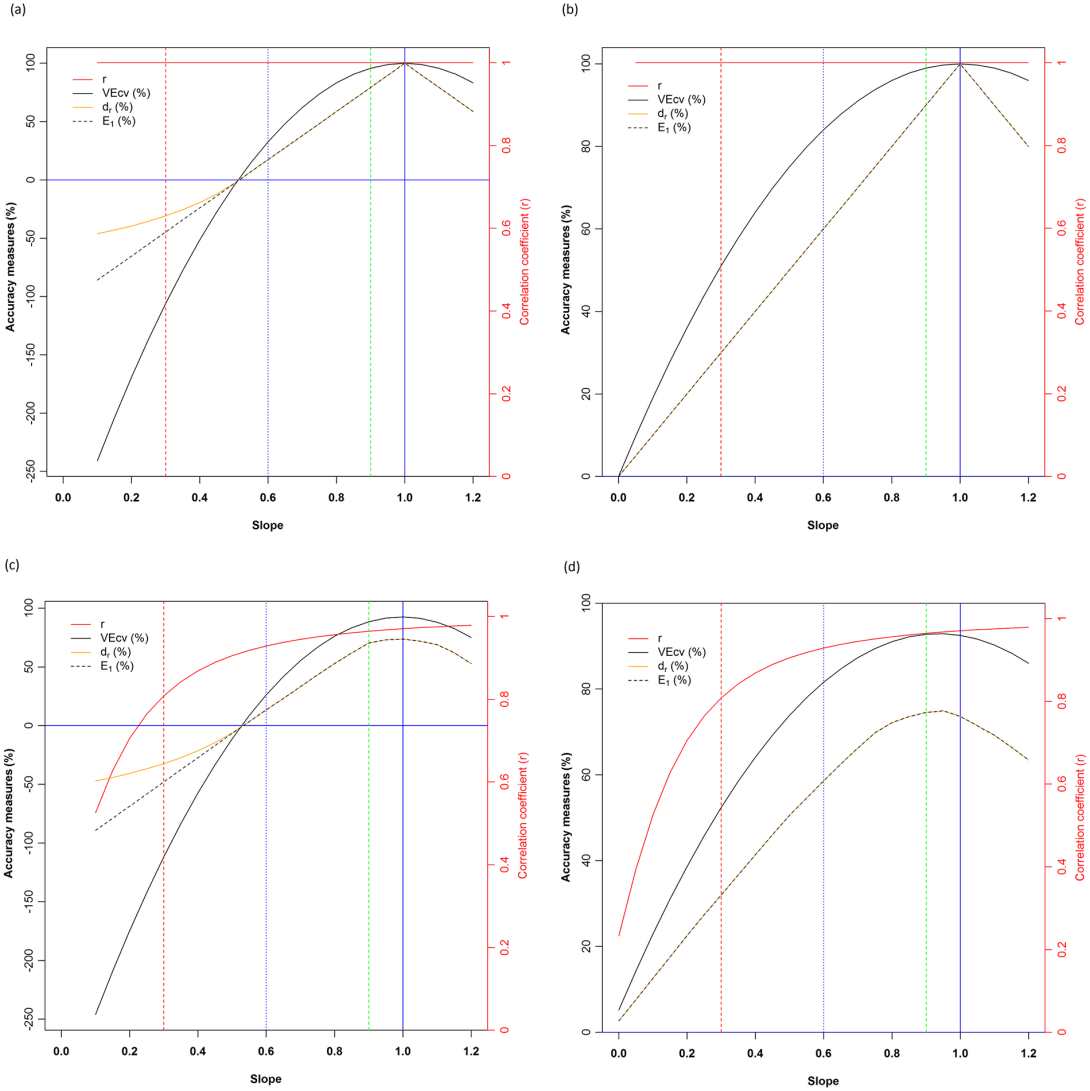
The changes of accuracy measures (*VEcv*, *d*_*r*_ and *E*_*1*_) and *r* with slope for the four simulated scenarios: a) scenario 1; b) scenario 2; c) scenario 3; and d) scenario 4. For each scenario, a slope of 1 (blue vertical line), 0.9 (green dashed vertical line), 0.6 (blue dashed vertical line) and 0.3 (red dashed vertical line), and *VEcv* = 0 (blue solid horizontal line) were highlighted.

For the first two scenarios (Panels A and B in S1 Fig), as expected, when y and x were equal or perfectly matched, *r* was 1 (Fig 2a and 2b). This indicated that *r* has correctly measured the matches when slope is 1. When the slope varied from 0.1 to 1.2 in both scenarios, it showed that *r* was constantly equal to 1 (Fig 2a and 2b), although it was expected to decline when the slope deviated from 1. Since the fitted values were used to calculate *r* as illustrated in Fig 1a and 1b, its value remained unchanged with slope in these scenarios (Fig 2a and 2b). Undoubtedly, *r* is misleading when the slope is not 1 in these two scenarios.

In contrast, *VEcv* was 100% as expected when the slope was 1 (Fig 2a and 2b). It declined when slope deviated from 1, dropping to 95.79%, 32.69%, -106.14% and -240.76% when slope was 0.9, 0.6, 0.3 and 0.1 respectively for the first scenario (Fig 2a) and diminishing to 99%, 84%, 51%, 9.75% and 0% when slope was 0.9, 0.6, 0.3, 0.05 and 0 respectively for the second scenario (Fig 2b). *VEcv* also declined when the slope was higher than 1 (Fig 2a and 2b). Since *VEcv* used the correct difference between the predicted and observed values as depicted in Fig 1, it has accounted for the changes in slope, has reflected such changes, and thus has reliably assessed the predictive accuracy.

As the slope deviated from 1, *VEcv* declines quadratically but *r* remained unchanged, showing that the bias (i.e., the departure of *r* from its corresponding *VEcv*) resulted from using *r* was getting increasingly larger (Fig 2a and 2b). The bias resulted from *r* was highlighted for slope at 0.3, 0.6, 0.9 and 1; and obviously the bias became increasingly larger when the slope further deviated from 1 in comparison with *VEcv*. This finding illustrated how *r* has failed to correctly measure the predictive accuracy and how misleading it is, so *r* cannot be used to assess predictive accuracy. It is also apparent that the *r* weighted by slope [3] also incorrectly reflects the predictive accuracy, although it indeed corrects some bias. This is because in the weighted *r*, the *r* was supposed to be linearly biased with slope, but the bias became quadratically higher when the slope deviated further from 1 (Fig 2a and 2b). Since *r* was constantly equal to 1, *r*^2^ would also be 1 and would display exactly the same pattern as *r*, thus *r*^2^ is misleading as well.

The last two scenarios (Panels C and D in S1 Fig) showed that *r* increased from 0.5252, 0.8083, 0.9288, 0.9643 to 0.9704 along slope ranging from 0.1, 0.3, 0.6, 0.9 to 1 for the third scenario (Fig 2c), and from 0.2328, 0.8083, 0.9288, 0.9643 to 0.9704 along slope ranging from 0, 0.3, 0.6, 0.9 to 1 for the fourth scenario (Fig 2d). Although *r* values declined as the slope became less than 1, its values were incorrect as they were based on the incorrect differences as illustrated in Fig 1. The change of *r* values with slopes in Fig 2c and 2d has revealed that it can even disguise its misleading behaviour because it indeed declined when the slope became less than 1. When the slope became larger than 1, the accuracy was expected to decline, but the *r* values, in fact, continued to increase from 0.9704 to 0.9788 for slope increasing from 1 to 1.2 for both scenarios (Fig 2c and 2d). This increase with the slope revealed the misleading behaviour of *r* when it is used to assess the predictive accuracy.

In contrast, for scenario 3, due to the noise in the predicted values, *VEcv* reached 92.51% when the slope was 1, declined when slope deviated from 1, dropped to 88.55%, 26.20%, -111.88% and -246.00% when slope was 0.9, 0.6, 0.3 and 0.1, and declined to 75.19% when slope was 1.2 (Fig 2c). For scenario 4, due to the noise in the predicted values, *VEcv* reached the maximum of 92.90% when slope was 0.95, decreased to 92.78%, 81.58%, 52.38%, 14.30% and 5.18% for slope at 0.9, 0.6, 0.3, 0.05 and 0 respectively, and declined to 85.98% for slope at 1.2 (Fig 2d). As discussed above, *VEcv* has correctly accounted for the changes in slope and thus has reliably assessed the predictive accuracy.

The changes in *r* values with slope in Fig 2c and 2d revealed that it also showed a similar trend as, and displays a correlation with, *VEcv*. This phenomenon can be found in previous studies [7]. This correlation could be because *r* is a component of *MSE* [7,17], hence a component of *VEcv* because *VEcv* can be expressed using *MSE* [18]. Such correlation may have contributed to the confusion about the suitability of r to assess predictive accuracy.

The bias resulted from using *r* was further depicted for slope at 0.3, 0.6, 0.9 and 1 in comparison with *VEcv* in Fig 2c and 2d. The bias became quadratically higher when the slope deviated from 1 instead of linearly, which is similar to what have been observed for the first two scenarios (Fig 2a and 2b). This finding demonstrated that the *r* weighted by slope [3] is also an incorrect measure of the predictive accuracy for the last two scenarios because the bias is non-linearly related to the slope.

For *r*^*2*^, it would display similar patterns as those displayed by *r* in Fig 2c and 2d.

**Fig 3:**
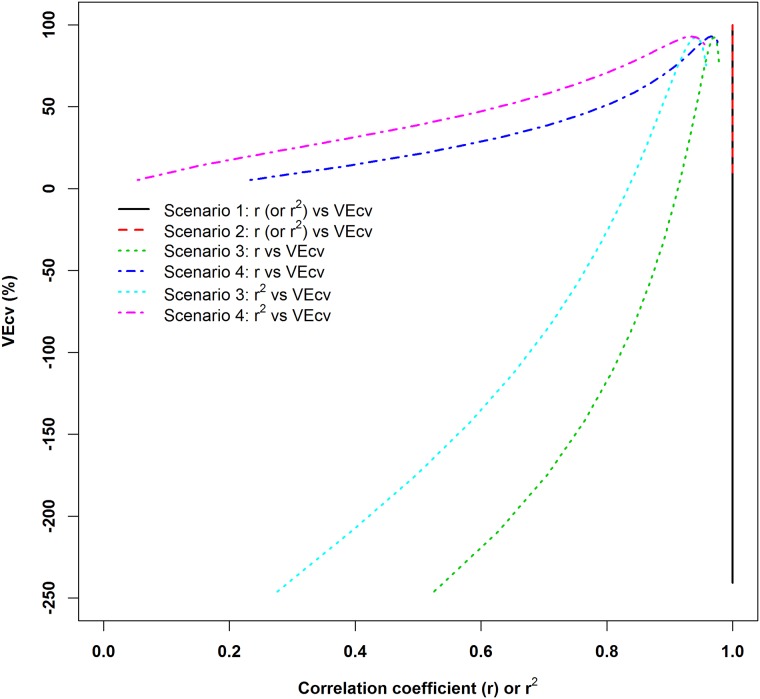
The relationships between *r* (or *r*^2^) and *VEcv* for the four simulated scenarios in Fig 1 and S1 Fig.

The relationships and mismatches between *r* and *VEcv* presented in Fig 2 were further depicted and highlighted in Fig 3. For *r*^*2*^, it also showed similar relationships with *VEcv* as *r* (Fig 3). The mismatches between *r* and *MSE* related measures were apparently present in previous studies (e.g., [7,34]), but no further action has been taken to investigate such phenomenon. Since *MSE* is linearly related to *VEcv* [18], *VEcv* would also be expected to have similar mismatches as in these previous studies, which are consistent with the findings of the current study. These mismatches suggest that the usual practice of comparing *r* and *r*^*2*^ values is also wrong because *r* and *r*^*2*^ with the same values can refer to different accuracy in terms of *VEcv* as shown in Fig 3.

Although these findings are based only on four simulated scenarios, they provide convincing evidence to support that *r*, the weighted *r* as well as *r*^*2*^ are incorrect measures of predictive accuracy.

### 3.3. Comparison of accuracy measures

The patterns of *VEcv*, *E*_*1*_ and *d*_*r*_ under four scenarios were displayed in Fig 2. It showed that *VEcv*, *E*_*1*_ and *d*_*r*_ all were 100% when the slope was 1; then they decreased when slope deviated from 1; *VEcv* declined quadratically while *E*_*1*_ and *d*_*r*_ decreased linearly, but they all reached 0% at the same slope for the first two scenarios (Fig 2a and 2b). When their values were > 0%, both *E*_*1*_ and *d*_*r*_ were identical. When their values were < 0%, *E*_*1*_ still decreased linearly with slope while *d*_*r*_ separated from *E*_*1*_ and decreased non-linearly with a slower pace than *E*_*1*_, and *VEcv* continued to decline quadratically with a faster pace than *E*_*1*_ (Fig 2a and 2b). The patterns displayed by *VEcv*, *E*_*1*_ and *d*_*r*_ in relation to slope for scenarios 3 and 4 were largely similar to those for the first two scenarios, although they all did not reached 100% due to noise in the data, with *E*_*1*_ and *d*_*r*_ decreased more than *VEcv* (Fig 2c and 2d). These findings support that 1) *d*_*r*_ is a linear rescaling of *E*_*1*_ when they are positive and 2) it is merely cosmetic and unnecessary to remap the negative values of *E*_*1*_ to *d*_*r*_ because a model with a negative *E*_*1*_ is flawed and of inefficacy and it is immaterial how it is scaled [31]. Moreover, the findings also suggest that the arguments and conclusion about *d*_*r*_ and *E*_*1*_ by Willmott et al. [30] are problematic because *E*_*1*_ and *d*_*r*_ were not identical in their study when they were non-negative. Thus only two accuracy measures, *VEcv* and *E*_*1*_, remain for further investigation.

It is clear that *VEcv* and *E*_*1*_ displayed monotonic changes relative to each other (Fig 2). The differences between *VEcv* and *E*_*1*_ in relation to slope were resulted from that *VEcv* was based on the square of the differences between the predictions for and the observations of validation samples while *E*_*1*_ was based on the absolute values of the differences. It demonstrates that both measures produced the same accuracy order for all predictive models under these four simulated scenarios. This finding suggests that *VEcv* and *E*_*1*_ are essentially the same in terms of relative predictive accuracy for the simulated scenarios, so the preference of *E*_*1*_ over measures based on the square differences by Legates and McCabe [8] is not supported under these simulated scenarios. This finding demonstrated that 1) the concern on the measure based on the square differences (i.e., *VEcv*) because it varies with the variability of the error magnitudes [8,30] is baseless; 2) both *VEcv* and *E*_*1*_ are equally interpretable. The key differences between them are that 1) *VEcv* explains the percentage of the variance of validation samples, while *E*_*1*_ explains the percentage of the sum of the absolute differences; and 2) *VEcv* is quadratically related to the differences between the predictions for and the observations of validation samples, while *E*_*1*_ is linearly related to the differences.

**Fig 4:**
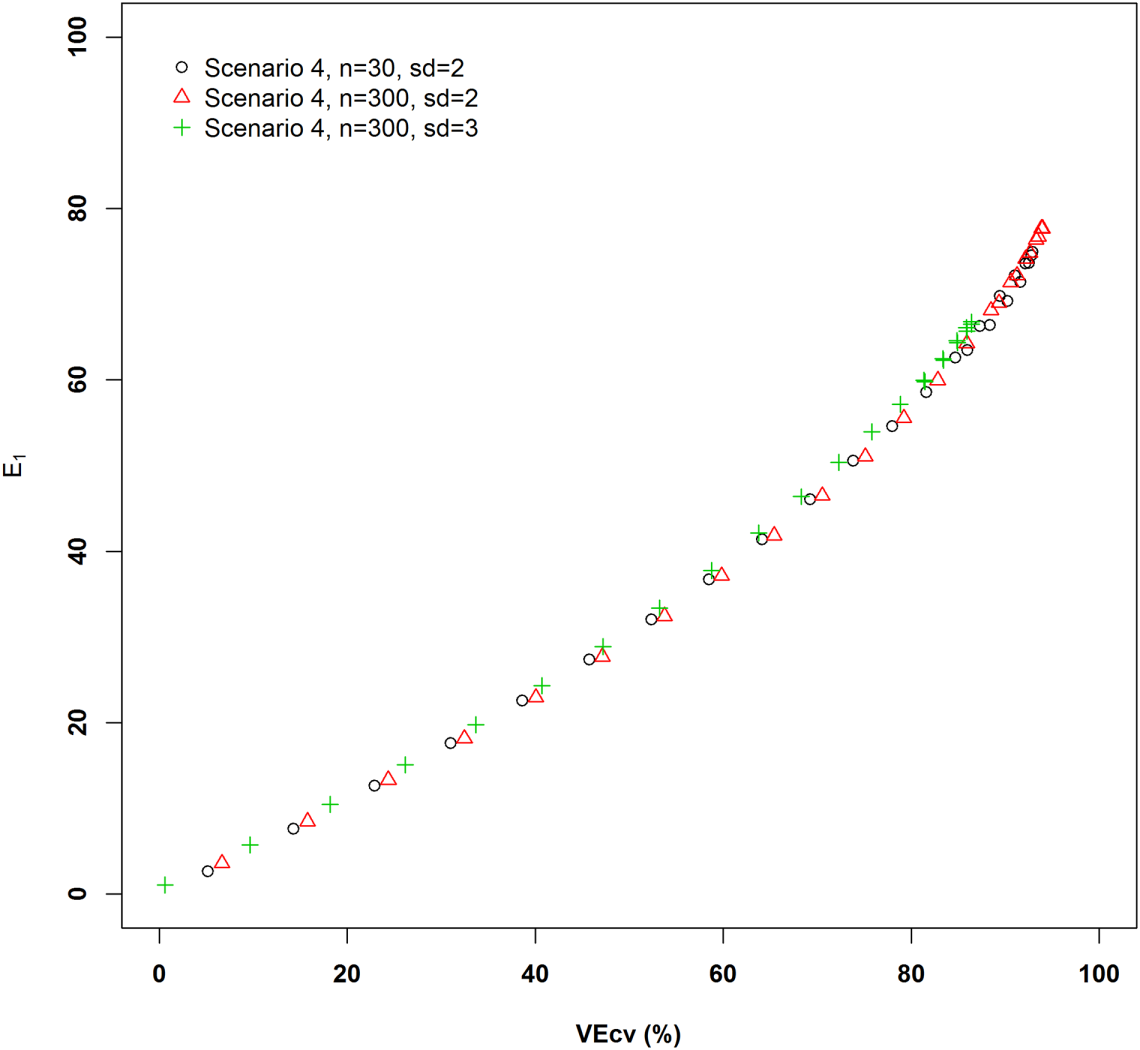
The relationships between *VEcv* and *E*_*1*_ for the fourth simulated scenario in S1 Fig and two additional extensions: 1) ε = *rnorm* (30, *sd* = 2), 2) ε = *rnorm* (300, *sd* = 2) and 3) ε = *rnorm* (300, *sd* = 3), with only the positive values presented.

The relationship of *VEcv* and *E*_*1*_ showed that they largely maintained the monotonic changes relative to each other, although some non-monotonic changes were displayed when different data noises were considered (Fig 4). It was concluded that *E*_*1*_ is preferred also because the measure based on the square differences varies with *E*_*1*_ but not monotonically [8,30]. This phenomenon is expected because for two datasets with the same *E*_*1*_ are not expected to be the same in terms of their data variation. On the other hand, it could also be stated as that ‘*E*_*1*_ varies with *VEcv* but not monotonically’. However, using either of them as a control to test the other requires solid justification that is lacking. The above findings actually suggest that *VEcv* and *E*_*1*_ should be used as complementary measures when one of them produces the same or similar accuracy values for predictive models, the other may be able to tell the difference between the models.

It is also apparent that the relationship of *VEcv* and *E*_*1*_ was maintained when the sample size increased from 30 to 300 for the same data variation and the standard deviation increased from 2 to 3 for the same sample size (Fig 4). This suggests that the relationship of *VEcv* and *E*_*1*_ is expected to be independent of sample size and data variation (i.e., error magnitude). These findings suggest that measures using the squared values do not respond differently to changes in sample size and data variation as measures using absolute values, which do not support the speculations on these issues by previous studies [8,22,23,30].

### 3.4. What should be used to assess the predictive accuracy?

Many measures for assessing the predictive accuracy have been reviewed or even recommended for numerical data [1,2]. Of these measures, besides *r* and *r*^*2*^, *MAE* and root *MSE* (*RMSE*) are among the most commonly used or recommended measures [1,2,6]. Therefore, the commonly used measures, *MAE* and *RMSE*, are considered in this study.

For *MAE* and *RMSE*, their advantages and disadvantages were discussed previously [22,23,35,36]. RMSE were criticised to suffer the following issues [22]: 1) it varies with the variability of the error magnitudes, 2) it varies with MAE but not monotonically, 3) its values are in between MAE and MAE* n^0.5^ (i.e., the square root of sample size n) and vary with (n^0.5^), and 4) it does not satisfy the triangle inequality of a metric. Of these issues, the first two issues are similar to what have been clarified above regarding the differences between *VEcv* and *E*_*1*_. As to the third one, it was clearly demonstrated that RMSE and MAE are highly linearly correlated according to the findings for relative *MAE* (*RMAE*) and relative *RMSE* (*RRMSE*) and has nothing to do with sample size n [18]. And for the fourth ‘issue’, it was proved to be not the case by Chai and Draxler [35]. Therefore, all these issues are largely invalid speculations. Furthermore, because the relationships between *VEcv* and *E*_*1*_ observed above and the relationships between RMSE and MAE [18], relevant arguments and speculations on the advantages and disadvantages of relevant measures using the squared or absolute differences need to be reassessed. Since all *MAE* and *MSE* related measures including *RMSE* as well as *VEcv* and *E*_*1*_ use the correct information to produce the predictive error or accuracy [18], they will be discussed below.

Of these *MAE* and *MSE* related measures, *MAE* and *RMSE* are the two most commonly used measures for assessing the predictive accuracy in the environmental sciences [6]. They are, however, unit/scale dependent (Table 2). Hence their application is limited to assessing predictive models that are applied to the same dataset. Moreover, they cannot tell how accurate the models are. The results based on these two measures for different datasets are not compatible, even for the same methods. This is because different datasets are usually different in unit/scale. According to Li [18], *MSE*, like *RMSE*, also shares these limitations.

**Table 2:** The relation of error/accuracy measures and data properties.

Error/accuracy Measure	Unit/scale independent	Variance-independent	Predictive accuracy	Relationship with VEcv*
*MAE*	No	No	Unknown	(1−VEcv/100)(n−1)2.0572ns
*RMSE*	No	No	Unknown	$\sqrt{(1-VEcv/100)(n-1)/n}s$
*MSE*	No	No	Unknown	$((1-VEcv/100)(n-1)/n)s^{2}$
*RMAE*	Yes	No	Unknown	(1−VEcv/100)(n−1)2.0572nCV
*RRMSE*	Yes	No	Unknown	$\sqrt{(1-VEcv/100)(n-1)/n}CV$
*SRMSE*	Yes	Yes	Unknown	$\sqrt{(1-VEcv/100)(n-1)/n}$
*MSRE*	Yes	Yes	Unknown	(1−VEcv/100)(n−1)/n
*VEcv*	Yes	Yes	Known	

* These equations were derived from Li [18], where *n* is the number of observations in, *s* is standard deviation of, and *CV* is coefficient of variation of, a validation dataset

*RMAE* and *RRMSE* are independent of unit/scale and not sensitive to data means according their definitions (Table 1). They enable us to compare results derived from different datasets that may have different unit/scale and different data means. However, they are linearly correlated with data variance [6,37]. Therefore, their application is limited to assessing predictive models that are applied to datasets with the same data variance which is hardly true in the reality. Furthermore, they are error measures and not accuracy measures, so they can only tell which model produce less error but they are unable to tell how accurate the models are. This may explain why there are so many published studies recommending models with negative *VEcv* to generate their predictions [18].

According to Li [18], standardised *RMSE* (*SRMSE*) and mean square reduced error (*MSRE*) don’t share the limitations associated with *RMSE*, but they are only error measures and still cannot tell the predictive accuracy as discussed above.

*VEcv* is an accuracy measure that is unit/scale, data mean and variance independent according to its definition [18]; and it unifies the error measures above via various equations in Table 2. It is an accuracy measure of predictive models and thus their predictions, and it provides a universal tool to assess and directly compare the accuracy of predictive models for any numerical data of various unit/scale, mean and variation from any disciplines. Moreover, these equations enable us to derive corresponding *VEcv* from relevant error measures and directly compare and assess the accuracy of predictive models for variables from different disciplines if relevant information is available as discussed previously [18].

Since both *VEcv* and *E*_*1*_ are reliable measures of the accuracy of predictive models, they could be used as complementary measures to each other as discussed in section 3.3. Hence they are recommended for future studies, although the relationships of *E*_*1*_ with the existing *MSE* and *MAE* related error measures are not as well defined as *VEcv*, which may be worth further investigation in future. With the applications of these accuracy measures, it would prevent flawed predictive models (i.e., models with negative *VEcv* and *E*_*1*_) be recommended to generate predictions. Consequently, predictive models with improved accuracy are expected to be developed to generate predictions for evidence-informed decision-making.

In addition, as demonstrated in previous studies, the randomness associated with cross-validation affects the accuracy measures [38–40], *VEcv* is thus also affected by such randomness [41]. So is *E*_*1*_ given that it uses the same information as *VEcv*. Therefore, we recommend that the cross-validation, with an exception of leave-one-out method, needs to be repeated a certain number of times (e.g., 100 times) to stabilise the *VEcv* and *E*_*1*_ in future studies. Furthermore, despite the assumption that *VEcv* and *E*_*1*_ were derived from validation results [8,18], they can be equally applicable to assessing predictive models based on new samples besides validation samples.

## Conclusions

This study has clarified relevant issues associated with predictive accuracy and predicted values. The calculation of *r* and *r*^*2*^ is not based on the difference between the observed and predicted values. They can only be used to assess the accuracy when predicted and observed values are perfectly matched; otherwise they do not measure the accuracy and are incorrect and quadratically biased. The weighted *r* is also incorrect measure of predictive accuracy. The usual practice of comparing *r* (and *r*^*2*^) values is problematic because *r* with the same values can refer to different predictive accuracy. The existing *MSE* and *MAE* related error measures suffer various limitations in their applications and are unable to tell the predictive accuracy. *VEcv*, an accuracy measure, unifies these error measures and is unit/scale, data mean and variance independent. It provides a universal tool to assess and directly compare the accuracy of predictive models for any numerical data of various unit/scale, mean and variation from any disciplines and is recommended for assessing the accuracy of predictive models in the future. Furthermore, *E*_*1*_ can be equally applicable to assessing the accuracy of predictive models as *VEcv*.

## Supporting information

S1 Fig Scenarios simulated for the relationship of the observed values (x) and predicted values (y) assumed to be linear with a slope of 1 (black line), 0.9 (green dashed line), 0.6 (blue dashed line) and 0.3 (red dashed line). **a**) x and y are perfectly linearly related, with an intercept of 0; **b**) x and y are perfectly linearly related, with intercepts changing with their associated slopes; **c**) x and y are linearly related, with certain noise (ε) in the predictions and with an intercept of 0; **d**) x and y are linearly related, with certain noise (ε) in the predictions and with intercepts changing with their associated slopes. The noise was randomly generated (i.e., ε = *rnorm* (30, *sd* = 2)).
(DOCX)
