# Supplementary material for: Assessing the accuracy of predictive models for numerical data: Not r nor r2, why not? Then what?
Source: PLoS One. 2017 Aug 24;12(8):e0183250. doi: 10.1371/journal.pone.0183250 (PMC5570302; doi:10.1371/journal.pone.0183250)

**Supporting Information 1**


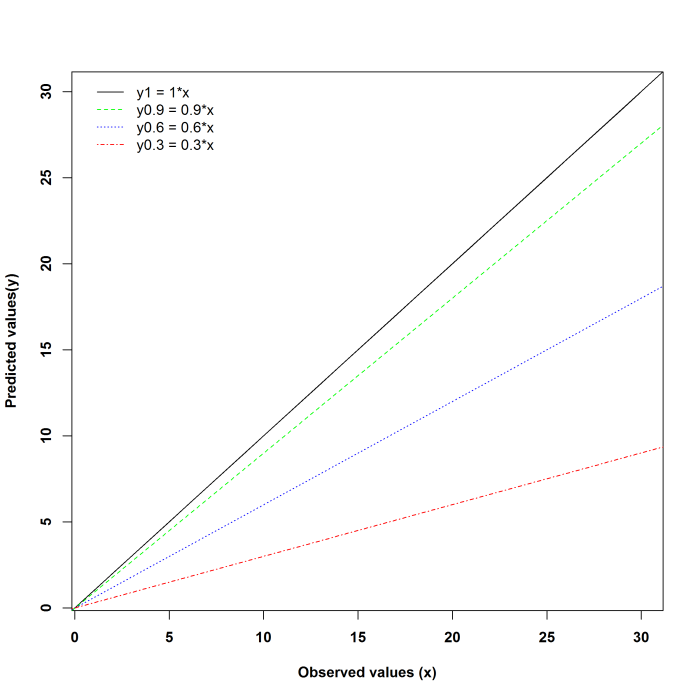


**(a)**


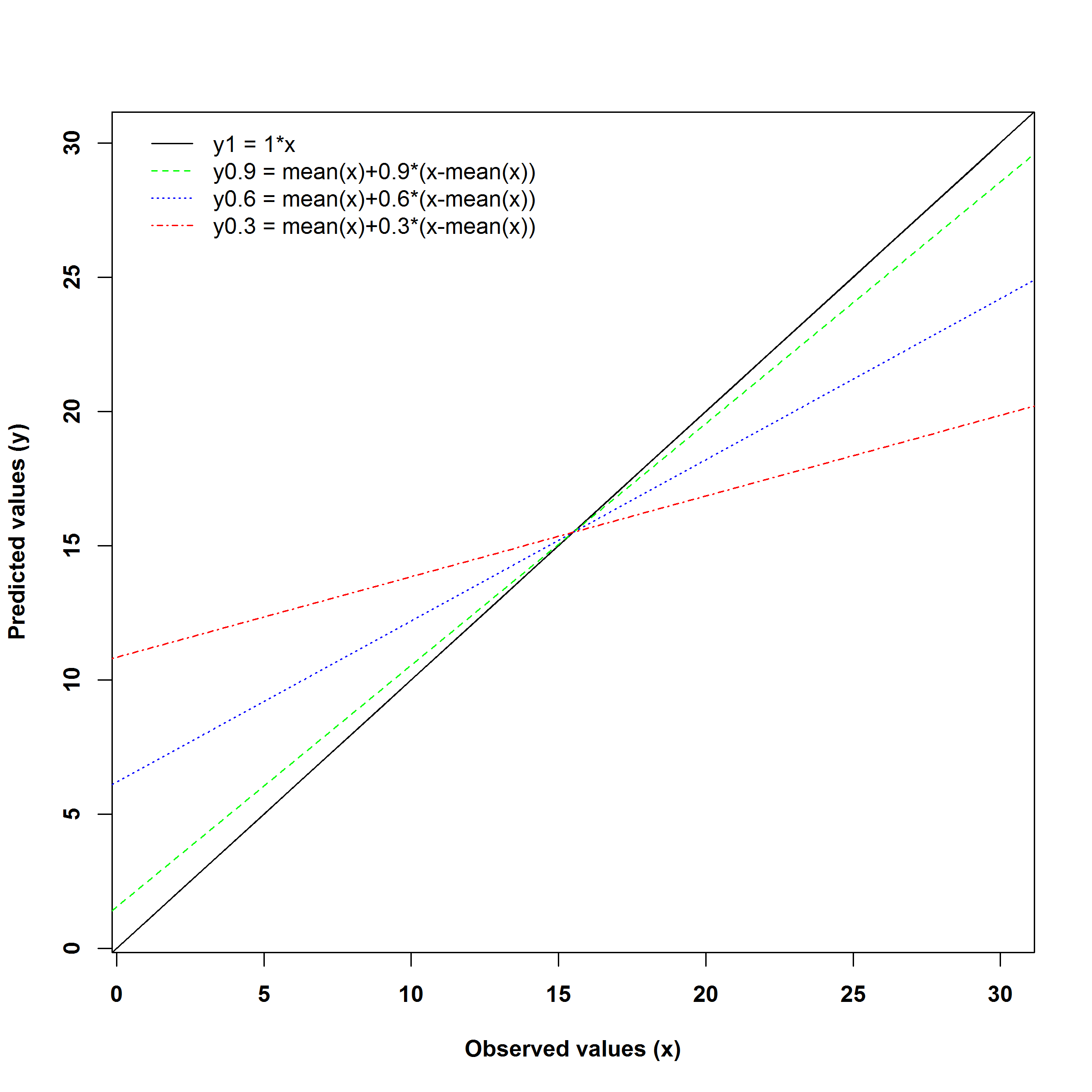


**(b)**

**Supporting Information 1.** Scenarios simulated for the relationship of the observed values (x) and predicted values (y) assumed to be linear with a slope of 1 (black line), 0.9 (green dashed line), 0.6 (blue dashed line) and 0.3 (red dashed line): **a**) x and y are perfectly linearly related, with an intercept of 0; **b**) x and y are perfectly linearly related, with intercepts changing with their associated slopes; **c**) x and y are linearly related, with certain noise (ε) in the predictions and with an intercept of 0; **d**) x and y are linearly related, with certain noise (ε) in the predictions and with intercepts changing with their associated slopes. The noise was randomly generated (i.e., ε = *rnorm* (30, *sd* = 2)).

**Supporting Information 1.** Cont.

**(c)**


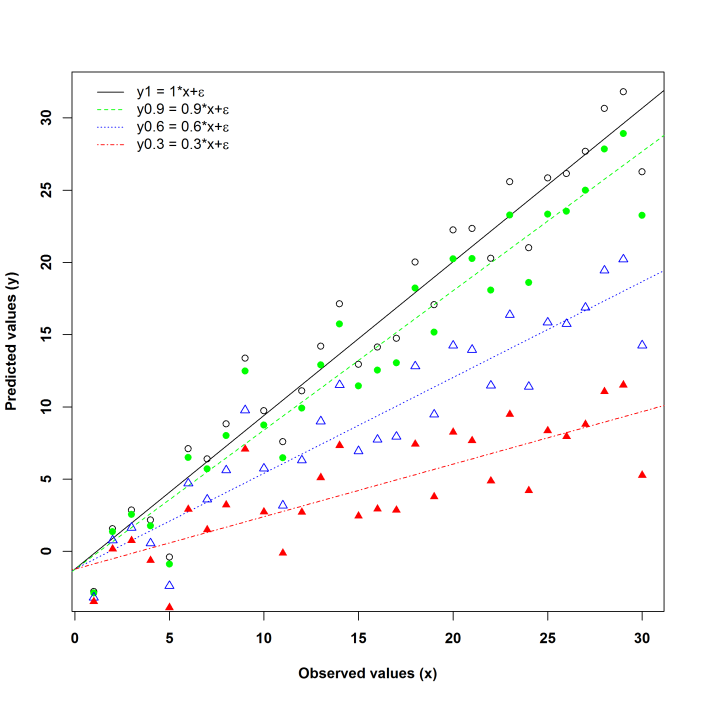


**(d)**


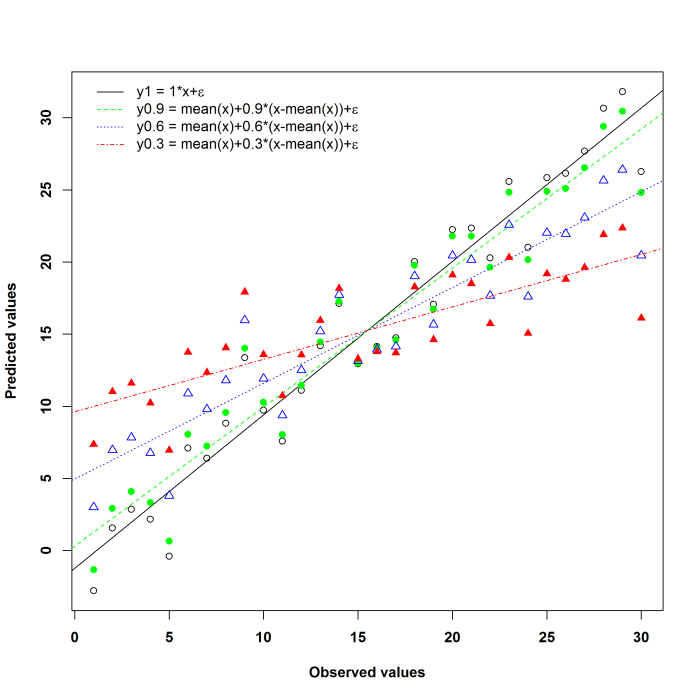

Supplement: S1 Fig — a) x and y are perfectly linearly related, with an intercept of 0; b) x and y are perfectly linearly related, with intercepts changing with their associated slopes; c) x and y are linearly related, with certain noise (ε) in the predictions and with an intercept of 0; d) x and y are linearly related, with certain noise (ε) in the predictions and with intercepts changing with their associated slopes. The noise was randomly generated (i.e., ε = rnorm (30, sd = 2)). (DOCX) [file pone.0183250.s001.docx]
